# Interactions among human papillomavirus proteins and host DNA repair factors differ during the viral life cycle and virus-induced tumorigenesis

**DOI:** 10.1128/msphere.00427-23

**Published:** 2023-10-18

**Authors:** Sebastian Wendel, Nicholas A. Wallace

**Affiliations:** 1 Kansas State University, Division of Biology, Manhattan, Kansas, USA; University of Michigan, Ann Arbor, Michigan, USA

**Keywords:** HPV, DNA repair, cervical cancer

## Abstract

This review focuses on the impact of human papillomavirus (HPV) oncogenes on DNA repair pathways with a particular focus on how these relationships change as productive HPV infections transition to malignant lesions. We made specific efforts to incorporate advances in the understanding of HPV and DNA damage repair over the last 4 years. We apologize for any articles that we missed in compiling this report.

## INTRODUCTION

Human papillomavirus (HPV) proteins manipulate the cellular DNA damage response (DDR) in the context of tumors as well as during the viral life cycle. HPV is a large family of viruses with double stranded circular DNA genomes of approximately 8 kilobases in size. HPVs are subdivided into the alpha-, beta-, gamma-, nu-, and mu-papillomaviruses based on sequence homologies in their L1 capsid protein (1, 2). Alpha-papillomaviruses are further sub-divided into “high-risk” (HR) and “low- risk” viruses based on the relative risk that an infection results in tumorigenesis. This review further specializes in recent advancements specific to high risk HPV with the rationale that there has been more research interest in these viruses, and as a result there have been more significant current advancements. Our one exception to this general guiding principle will be a brief discussion of beta-papillomaviruses because there have been several notable advancements regarding the manipulation of DDR by these viruses.

For some time, the ability of HR alpha-papillomaviruses (referred to in this review as HPV, for simplicity’s sake) to promote carcinogenesis has been understood at a molecular level of detail. These HPVs express two primary viral oncogenes (HPV E6 and E7) that disrupt major cellular tumor suppressor pathways. HPV E6 forms a complex with p53 and a cellular ubiquitin ligase, E6AP. This allows HPV E6 to promote the ubiquitination and subsequent degradation of p53 (3–5). HPV E7 dysregulates cell cycle progression by binding and destabilizing RB and RB family proteins (6, 7). The most immediate impact of the HPV E7-induced destabilization of RB and RB family proteins is increased cellular replication stress, via abrogation of the primary G1/S checkpoint (8). In turn, cells respond to E7-induced replication stress by changing the expression of demethylases that allow them to tolerate the increased replication demands (9, 10). While these classical observations provide a strong basis for understanding how HPV transforms cells, the virus also promotes the aberrant activation of DNA repair responses to facilitate its life cycle. As proper functioning of the DNA repair response protects the host genome from accumulating mutations, it is also an important tumor suppression mechanism. Therefore, this review will discuss recent advancements in understanding how HPV influences DNA repair in the context of the HPV life cycle and in HPV-associated tumorigenesis.

### A brief introduction to DNA damage repair

While DNA damage is frequently referred to as a type of singular homogeneous challenge to genome stability, there is a significant diversity in mutagenic risk of damage and sources of DNA damage. DNA can be damaged by ultraviolet light from the sun, oxygen radicals produced during metabolism, x-rays, gamma rays, chemical carcinogens, and physical stress during replication, among a litany of other endogenous and exogenous causes. Broadly speaking, types of damage can be grouped into three categories: (i) bases missing from the backbone or crosslinked to other bases, (ii) a single strand break in the DNA backbone, or (iii) a double strand break in the DNA backbone or DSB. The mutagenic risk of these lesions varies within the groups. For example, the mutations that arise from the misrepair of a damaged base are less severe than those caused by a misrepaired DSB (11). The cell cycle position of the cell trying to repair these damages also dictates their mutagenic potential as well as the cellular response that will be employed to repair the damage. Although there is some overlap among them, there are DNA repair pathways that specialize in each type of DNA damage at different points in the cell cycle. A recent and thorough review of DSB repair discusses these topics in more detail (12).

There are also a variety of different types of mutations that can arise from DNA damage. This includes single and double nucleotide changes, insertions (inverted or otherwise), deletions, tandem duplications, and others. Insertions, deletions, and tandem duplications can be large or small (13). Nucleotide changes can be grouped based on where they occur (e.g., at adenosines between thymines) and or what base they substitute for (e.g., replacing a cytosine). A somewhat recent and rapidly expanding aspect of DNA repair research has been the generation and detection of mutational signatures, where the variety of mutations in a tumor is matched to a particular source of DNA damage (14). Probably the most famous signature is the C→T and CC→TT mutations associated with UV exposure (15).

Replication stress is a major endogenous source of DNA damage (16). It is also one of if not the most relevant source to consider with cervical cancer, CaCx (17–19) (APOBEC-driven mutations are also very common in CaCx). Because of its importance and also the dizzying diversity of DNA repair responses and the focus of this review, we will only discuss the cellular responses that are likely induced by HPV E7-associated replication stress (8). Replication stress is a collective term used to describe any change in a cell that delays the activity of replicative polymerases (20). This can include physical barriers like those caused by UV radiation or, as is the case with HPV E7-associated replication stress, a suboptimal supply of nucleosides. The primary risk of replication stress is not the delay itself but rather the decoupling of the replicative helicase from the polymerase. The helicase continues unimpeded by the barriers to polymerase activity, resulting in long unstable stretches of single stranded DNA. Binding of RPA trimers to this single strand DNA helps stabilize the single stranded DNA (21). RPA binding also initiates the translesion synthesis (TLS) pathway by activating the RAD6/RAD18 complex which ubiquinates the clamp (proliferating cell nuclear antigen or PCNA) that holds polymerases at the replication fork (22). The ubiquitination shifts the binding affinity of PCNA from favoring replicative polymerases to favoring TLS-specific polymerases (e.g., POLη). Unlike replicative polymerases, TLS polymerases can add untemplated bases allowing them to bypass most sources of replication stress. This allows DNA synthesis to catch up with the helicase, turning off the TLS pathway, and switching the binding affinity of PCNA back to favoring replicative polymerases. While TLS polymerases are by nature more mutagenic than replicative polymerases, they prevent replication stress from causing DSBs via replication fork collapse (22).

For this reason, TLS is often described as a DNA damage tolerance (rather than repair) pathway. When the TLS pathway fails, the resulting DSB is most likely to be repaired by the homologous recombination (HR) pathway which is most active during the S and G2 phases of the cell cycle (23). The cell cycle preference of the HR pathway is due to its reliance on a homologous DNA template to repair DSBs. Briefly, HR proteins promote DNA resection on either side of the lesion (24). BRCA1, PALB2, and BRCA2 facilitate the coating of the resulting single stranded DNA with RAD51 filaments. Once loaded onto single stranded DNA, RAD51 finalizes the repair by performing a search for homologous DNA (and strand invasion) that can be used as a template for high fidelity repair.

### HPV is a prolific infectious agent and deadly carcinogen

The estimated lifetime probability of having had at least one HPV infection is nearly 85% for women and over 90% for men (25). Thus, the virus is clearly prolific. These infections cause about 5% of all cancers worldwide and kill hundreds of thousands of people each year (26). Although this is clearly a monumental amount of death and disease, it is equally obvious that despite the “high-risk” moniker, causing cancer is not a normal part of the HPV life cycle. In fact, one of the most common changes that occurs to HPV as its host cell undergoes transformation (integration of the viral genome) prevents production of infectious viral particles. The HPV genome is circular, meaning it must linearize to insert into the human genome. The HPV genome is also dense with overlapping open reading frames and minimal intergenic spaces where linearizing could be accomplished without disrupting expression of an HPV gene. There appears to be a selection for disrupting the E2 gene in HPV+ cancers, likely because HPV E2 acts as a negative regulator of HPV oncogene expression. This makes HPV gene expression different during the HPV life cycle compared to what it is in HPV+ cancers. Given that one of the most dominant changes during HPV-associated carcinogenesis is increased HPV oncogene expression and that HPV oncogenes cause major alterations in the cellular DDR response, this review will distinguish between these two phases of HPV-host interactions.

### Manipulation of DDR by HPV differs between productive infection and tumorigenesis

The HPV life cycle has three distinct parts. During viral establishment, infectious viral particles encounter basal epithelial cells and traffic to the nucleus via the Golgi. There have been excellent recent reviews on HPV trafficking that we are not qualified to expand on (27–29). Once establishing an infection, HPV begins the maintenance phase of its life cycle, where the virus is maintained in cells at a relatively low copy number. During maintenance, HPV replication is limited to levels that allow it to double along with the basal epithelial cells expanding laterally. The third portion of the HPV life cycle is known as amplification and is most heavily reliant on the manipulation of host DNA damage responses. In this phase of the HPV life cycle, the viral copy number per cell is frequently an order of magnitude higher than during the maintenance phase. Viral replication and HPV oncogene expression are at their highest. Prior reviews provide an extensive chronicling of these interactions (30–32) and a more recent review discusses the HPV life cycle in the context of innate immunity (33). In this section, we aim to highlight the most recent findings relevant to the manipulation of the host genome by HPV and thus focus primarily on the amplification phase of the HPV life cycle.

The Laimins, Fradet-Turcott, and Moody labs have identified multiple components of the ATM and ATR DNA damage sensing pathways as well as the homologous recombination DSB repair pathway that are required for HPV amplification (34–37). Indeed, these signaling pathways become hyperactivated during amplification resulting in considerable changes in the cellular environment. Recent work has provided a deeper mechanistic understanding of how HPV oncogenes activate these DNA damage sensing pathways and result in further downstream impact. For example, the ability of HPV E7 to target a cellular E3 ubiquitin ligase, RNF168, is critical for manipulating the DNA repair response (38). Furthermore, some of the DNA damage signaling caused by HPV is the result of topoisomerase 2β-induced DSBs that help promote HPV replication (39). Because cells are invested so heavily in protecting their genome from DNA damage, the manipulation of the DNA damage response by HPV also allows the virus to alter pathways adjacent to these responses. Specifically, HPV alters p62-mediated autophagy via its influence on ATR (40).

For some time, the activation of DNA repair responses by HPV has been linked to the induction of replication stress by HPV E7, particularly through the depletion of nucleoside pools (8, 41). This position is solidly supported by the observation of replication stress markers, but perhaps stronger evidence was obtained by showing that cells adapted to HPV E7 expression by becoming reliant on mechanisms that increase nucleoside pools (9, 10, 42). However, as noted in our overview of the cellular response to replication stress, the TLS pathway is designed to prevent replication stress from resulting in the DSBs associated with the expression of HPV oncogenes. Whether HPV oncogenes were directly contributing to replication stress induced DSBs by blocking TLS or indirectly by causing more replication stress than the pathway could manage was unclear. Recent work demonstrated that both HPV oncogenes limit the ability of the TLS pathway to protect against replication stress. HPV E7 primarily acts by taxing the TLS pathway via high levels of replication stress leaving it less capable of dealing with replication stress from other sources (43). HPV E6 is more directly involved in impairing TLS, through its destabilization of p53. p53 is a transcription factor for the TLS-specific polymerase POLη (44–46) and possibly other TLS-specific polymerases (e.g., POLι and POLκ) (44). HPV E6 does not attenuate basal POLη expression but rather prevents its induction in reaction to exogenous sources of replication stress, including clinically relevant drugs like cisplatin or HPV E7 expression (44). Both the activation of the TLS pathway and induction of replication stress are dependent on HPV E6 and E7 functions, p53 and RB destabilization, respectively (43, 47). Thus, they both seem to be characteristic features of high-risk HPVs.

Despite the induction of DNA repair responses and DNA damage, the HPV genome is not prone to rapid mutations like other viruses. The interactions between its core replication proteins seem at least partially responsible. HPV E1, likely through its helicase activity, activates DDR. This activation appears localized to HPV replication foci, where it acts along with HPV E2 to produce viral replication factories that include DNA damage repair factors (48). Among the proteins recruited to these viral factories, TOPBP1 appears central to the maintenance of HPV genomic integrity. TOPBP1 is a cellular protein involved in multiple DNA repair and signaling pathways, including the replication stress response that activates the TLS pathway (49). The extent to which the TLS pathway contributes to HPV replication is unclear but is supported by the observation that TOPBP1 facilitates ATR activation (promotes TLS by promoting POLɳ stabilization) during the viral life cycle (50). Regardless of this ambiguity, the interaction between the E2 protein from HPV16 and TOPBP1 is required for viral genome stability (51) and requires the phosphorylation of HPV E2 by the cellular CK2 kinase (52). HPV E2 is also responsible for preventing activation of the DNA damage response from causing senescence (53).

During the viral life cycle, HPV E6 and E7 serve to manipulate the host cellular environment to make it more conducive to HPV replication. One of the ways that they do this is to activate DNA repair responses, as noted in the preceding section. They also actively move DNA repair factors away from the host genome and to sites of viral replication during the amplification stage of the HPV lifecycle (54). During tumorigenesis, HPV oncogenes are as highly expressed as they might be during amplification, but there is most often no HPV replication. The lack of a place to bring the proteins does not prevent HPV oncogenes from moving DNA repair factors away from damaged cellular DNA (54, 55). This impedes multiple DNA repair pathways, including mislocalization of FANCD2, which impairs the Fanconi anemia pathway that fixes DNA crosslinks during S-Phase (56, 57). HPV oncogenes similarly inhibit repair of DSBs by the homologous recombination pathway (55). As expected, HPV oncogenes repair DSBs using the microhomology mediated end joining pathway, a repair pathway that is employed when homologous recombination cannot be completed (58). There is also a growing body of evidence that HPV oncogenes dysregulate non-coding RNAs to modulate DNA repair responses (59). This includes suppression of the p53-regulated long non-coding RNA, damaged induced non-coding RNA, or DINO expression in HPV+ CaCx (60). The decreased DINO abundance plays a role in HPV-mediated repression of the ATM/CHK2/p53 signaling cascade in HPV transformed cells. Whether there are differences between DINO-mediated inhibition of p53 between HPV transformed tumors and cells with productive HPV infections is unclear.

### There are potential therapeutic benefits to the understanding how HPV alters DNA repair

**Fig 1:**
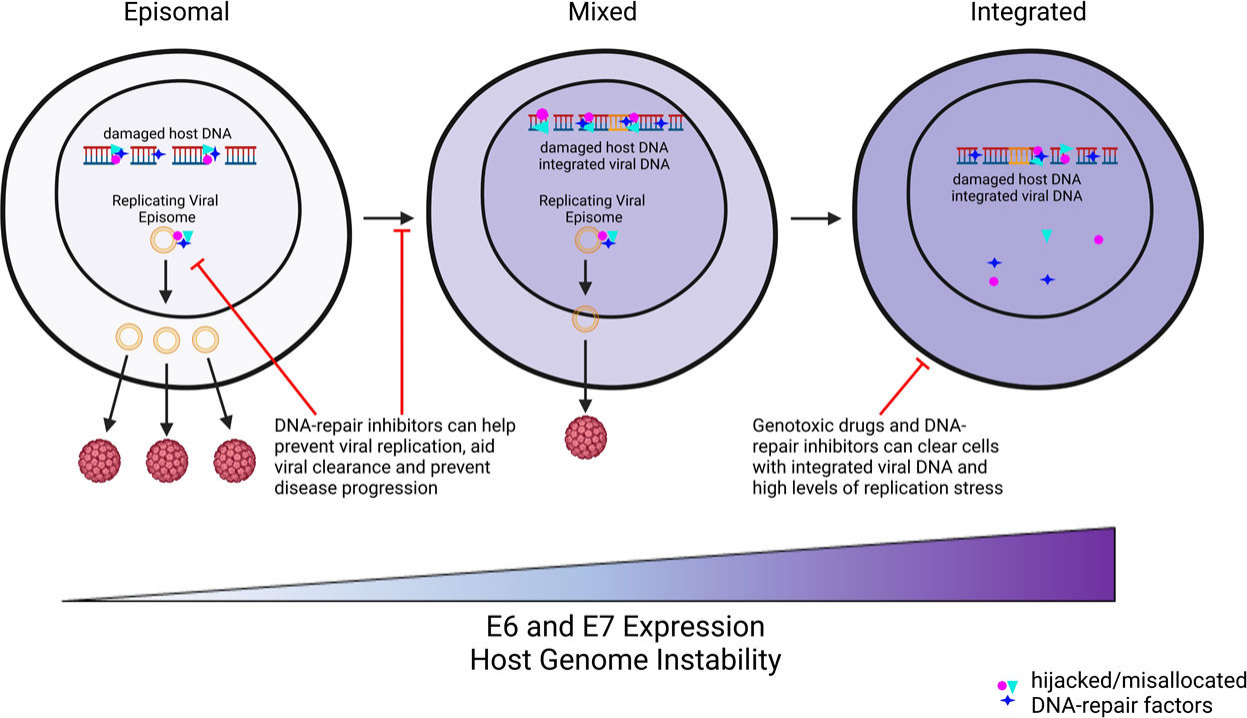
Therapeutic windows based on viral genome integration status. During a productive HPV infection, HPV oncogenes move repair factors from damaged cellular DNA to sites of viral replication. These factors are required for HPV amplification. When integration of the HPV genome occurs during tumorigenesis, HPV is no longer able to replicate, but repair factors are still moved away from damaged cellular DNA. We propose that this results in different therapeutic opportunities based on the physical status of the HPV genome. When the viral genome is episomal, inhibition of host DNA-repair factors localized to viral replication centers is likely to promote clearance by impeding viral genome amplification. When the viral genome has integrated, DNA repair foci indicate repair factors that are unavailable to repair damage to cellular DNA (i.e., a repair deficiency) that could be exploited to treat tumors by causing the types of damage repaired by these factors. It is unclear if DNA repair inhibitors or additional DNA damage would hinder viral replication, prevent progression of premalignant lesions, promote integration of the HPV genome by increasing the number of double stranded DNA breaks, or some combination of these. Created with BioRender.com.

One of the reasons for noting the differences in HPV’s manipulation of DNA repair during the viral lifecycle compared to virus-mediated transformation is that the therapeutic advantages of this knowledge also differ in each situation. Figure 1 illustrates the different potential therapeutic windows depending on whether the viral genome is episomal, integrated, or both. During the viral lifecycle and from the perspective of viral propagation, HPV oncogenes typically activate DNA repair machinery and bring those factors to the replicating viral genome. This knowledge suggests that inhibiting ATM or ATR signaling or the homologous recombination pathway would impede viral replication. When considered from the perspective of genome integrity in an HPV transformed cell, HPV impairs DNA repair pathways by causing damage (collapsed replication forks) and then preventing the repair machinery that would respond from optimally localizing to the lesion. In the case of HPV+ tumors, inhibiting the ATM or ATR signaling pathway might have no impact or even enhance the HPV-induced genome instability that contributes to tumorigenesis.

In this light, it is interesting to think about DNA repair protein abundance as a biomarker for HPV associated disease. Our group and others have already taken a step toward using HPV-oncogene driven DDR expression changes as biomarkers. Specifically, separate studies have shown that the expression of DNA repair factors correlates with CIN progression and can distinguish between HPV+ and HPV− head and neck cancers (44, 61, 62). Moving forward, the key will be to develop a more exact understanding of how to use these expression changes to guide care. Does detecting a high abundance of homologous end joining proteins in an HPV+ tumor in infection counterintuitively indicate that the tumor has defective homologous recombination? If so, it might be used as a marker of a tumor with a “BRCAness” phenotype that would as a result be responsive to PARP1 inhibition. There is at least some indication that this could be the case as a recent study demonstrated that CaCx can be successfully treated with the PARP1 inhibitor Olaparib (63).

### Beta-genus human papillomaviruses also impair the DNA damage response: will we ever know if it matters for the general population?

The review to this point has intentionally only focused on a small subset of a single genus of papillomaviruses, the high-risk α-HPVs. However, members of the beta-papillomavirus genus (or β-HPVs) also alter DNA repair signaling (64–66). Unlike high-risk α-HPVs, β-HPVs infect the skin typically outside of the genital tract. Because β-HPV infections typically occur in sun exposed areas, their primary motivation for interacting with DNA repair factors appears to be blocking the cell cycle arrest that would otherwise accompany UV damage. In one way this is similar to high-risk α-HPVs, as the goal is to propagate the viral life cycle. However, β-HPV does not appear to undergo a robust amplification stage in their lifecycle and do not seem to require DNA repair factors in high abundance to protect viral genome fidelity.

β-HPV infections are also less closely connected to tumorigenesis and do not persist in tumors. Thus, β-HPV infections definitively do not result in tumors that require persistent viral gene expression like high-risk α-human papillomaviruses. Instead, β-HPV infections have been proposed to promote genome destabilization indirectly by impairing the cellular DNA damage repair responses, leaving mutations that drive tumorigenesis without the direct contribution of continued viral gene expression. The evidence for this so-called “hit and run” mechanism is mixed, and a “smoking gun” has been difficult to identify. Infections with a subset of β-HPV are associated with non-melanoma skin cancers in people with EVER1 and EVER2 mutations that cause an immune defect that somewhat specifically results in persistent β-HPV infections (67). These tumors appear in sun exposed regions of the body supporting the complementary (rather than dominant) role of the virus in tumorigenesis. Similarly, other people experiencing more generalized immune suppression (e.g., medically induced immune suppression after organ transplantation) also develop cancerous skin lesions that appear to be linked to β-HPV infections.

The difficulty in connecting β-HPV infections with squamous and basal cell cancers begins with the efforts needed to determine if the observations in people with impaired immune systems extend to the general population. β-HPV infections are widespread and often occur early in childhood. Unlike α-HPV infections, they are spread by skin-to-skin contact, and thus there is no surrogate measure to identify people (e.g., people prior to sexual debut) who are unlikely to have been exposed to the virus. When efforts have been made to connect serological data indicating prior exposure to β-HPV infections with risk of skin cancer, modest odds ratios are observed (68). Furthermore, the β-HPV infections that are associated with skin cancer in people with EVER1 or EVER2 mutations do not seem notably more closely associated with tumorigenesis in the general population (68). Of course, this does not provide definitive evidence that β-HPV infections do not promote the acquisition of tumorigenic mutations present in skin cancers.

The most consistently positive aspect of research into β-HPV has been in the sphere of basic sciences. For example, for some time now, it has been understood that the E6 from the β-HPVs linked to skin cancers in people with EVER1 and EVER2 mutations have a profound ability to impede cellular DNA repair. The molecular details have also been known, as these β-HPV E6 act by binding and destabilizing the cellular histone acetyltransferase, p300. This leads to reduced expression of a handful of essential DNA repair genes. The limited availability of these repair factors increases the chance that UV damage causes a DSB and further prevents the error free repair of these lesions via the homologous recombination pathway (69–71). Perplexingly, despite inhibition of a major DSB repair mechanism, β-HPV E6 only delays, rather than blocks, DSB repair (71).

More recent work has addressed the previously lingering question of how DSBs are repaired in the presence of β-HPV E6. Repair of the DSB by non-homologous end joining pathway, which becomes more active when homologous recombination becomes impaired, seemed like the most likely answer until β-HPV E6 was shown to impair this pathway as well (72). β-HPV E6 disrupts both pathways after they have begun causing repair complexes to form but prevents their resolution. β-HPV E6 then promotes the colocalization of non-homologous end joining and homologous repair factors that then act in what appears to be a novel DSB repair mechanism (73). β-HPV E6 also promotes the use of an emerging mutagenic DSB repair pathway known as alternative end joining or microhomology mediated end joining (74) .

Other β-HPV proteins also hinder the DNA repair response. For example, the recent observation that both β-HPV E6 and E7 downregulate expression of CHK1, a DNA damage sensing kinase (75), is notable. CHK1 is part of the ATR signaling cascade, which as discussed above is impaired by β-HPV E6. The fact that a virus with such a limited coding potential would commit to these overlapping strategies to impair this signaling response suggests that the ATR/CHK1 signaling pathway is a strong negative regulator of the β-HPV life cycle. Few labs in the world have studied β-HPV replication and thus are equipped to test this hypothesis and if pertinent how broadly (across the genus) ATR/CHK1 signaling serves as a β-HPV restriction factor. Among them, the Stubenrauch lab is probably most able to do this work in part thanks to their recent excellent paper where they demonstrate that E8 and E2 fusion protein from HPV49 represses viral genome replication and as a result prevents HPV49 from immortalizing keratinoocytes (76).

There is an array of animal models that provide insight into β-HPV biology and pathogenesis. These include transgenic systems where mice express β-HPV E6 and E7 or the complete early region of the β-HPV genome driven by keratin gene promoters. One notable system flanked β-HPV E6 and E7 with LoxP recombinase sites and removed the viral genes once a tumor had formed to demonstrate that β-HPV oncogenes can cause cancers that do not require continued viral gene expression (77). There are also papillomaviruses that infect model organisms and provide insight into β-HPV infections in humans. The two primary viruses used in this work are Mastomys natalensis papillomavirus (MnPV) and Mus musculus papillomavirus 1 (MmuPV1) which naturally infect *Mastomys natalensis* and *Mus musculus,* respectively. Both of these systems have been used recently to make exciting advances in the field, most notably obtaining details of MmuPV1 transmission between mice, demonstrating that vaccinations against MnPV can protect against infection and the development of a system to track MmuPV1 infected cells and their progeny via fluorescence-based tools (78–80).

In general, the evidence from countless studies suggests that β-HPVs have characteristics consistent with the idea that they might play a role in skin cancer initiation (81). However, there was a single study that used the MMuPV1 model to suggest that β-HPV infections might actually protect against skin cancer (82). Notably, this group was unable to reproduce results that have been obtained by multiple other labs using the same virus and to our knowledge their results have not since been independently reproduced. The stronger opposition to the idea that β-HPV infections play a role in skin cancer development comes from within the field and does not question that these viruses might play a role in tumorigenesis. Their reasonable criticism is that after decades of investigation no one has found a “smoking gun” connecting β-HPV infections to skin cancer and that they feel there is currently no viable strategy for providing one. A counterargument to this position is that a wide array of mutational signatures has been defined that allow mutations in tumors to be linked to the mutagens that created them (14). There is no *a priori* reason that a signature for β-HPV associated mutations could not be defined and used similarly.

### Four outstanding questions

This review will close with four questions related to HPV and DNA repair that may be important to consider as the field moves forward.

1. Can biomarkers be identified to triage patients with cervical dysplasia who are at increased risk of progression?
2. Can the dependency of HPV infections on the DNA repair response be leveraged to produce antivirals that can prevent HPV infections from progressing toward tumorigenesis?
3. Do the defects in DNA repair associate with HPV oncogene expression in tumors (i.e., in the absence of active viral propagation) result in synthetic lethal relationships that can be exploited via targeted therapies?
4. Will a smoking gun connecting β-HPV infections to skin cancer be found or will the field begin to focus on other aspects of β-HPV biology?
